# A year of Covid-19: experiences and lessons learnt by small European island states—Cyprus, Iceland and Malta

**DOI:** 10.1093/eurpub/ckab217

**Published:** 2022-01-03

**Authors:** Sarah Cuschieri, Elena Pallari, Amalia Hatziyianni, Rannveig Sigurvinsdottir, Inga Dora Sigfusdottir, Árún Kristín Sigurðardóttir

**Affiliations:** 1 Department of Anatomy, Faculty of Medicine and Surgery, University of Malta, Msida, Malta; 2 MRC Clinical Trials and Methodology Unit, University College London, London, England; 3 Ammochostos General Hospital, Paralimni, Cyprus; 4 Department of Psychology, Reykjavik University, Reykjavik, Iceland; 5 Teacher’s College, Columbia University, New York, NY, USA; 6 Icelandic Centre for Social Research and Analysis (ICSRA), Reykjavik, Iceland; 7 School of Health Science, University of Akureyri, Sólborg, Iceland; 8 Akureyri Hospital, Akureyri, Iceland

## Abstract

**Background:**

COVID-19 became a global pandemic within weeks, as every country including small states and islands experienced a surge in cases. Small islands are known to face several challenges in the quest to curb the viral spread, but with the absence of land boarders and small population size, these factors should have played to their advantage to minimize the spread. The aim of this article was to compare and contrast the COVID-19 situation, restrictions, preparedness, management and the healthcare systems between the small population island states of Cyprus, Iceland and Malta.

**Methods:**

Data were obtained from Ministry of Health websites and COVID dashboards of the three respective Island states in Europe. Comparisons were made between the reported cases, deaths, excess deaths, years of life lost, swabbing rates, restrictive measures, vaccination roll-out and healthcare system structures.

**Results:**

Cyprus and Malta contained the COVID-19 spread better than Iceland during the first wave. However, a significantly higher viral spread and mortality rates were observed in Malta during the second waves. Similar healthcare preparedness and services, restrictions and relaxation measures were implemented across the three islands with some exceptions. Covid-19 vaccination has initiated across all Islands with Malta leading the vaccination roll-out.

**Conclusion:**

The small population size and island status proved to be an asset during the first wave of COVID-19, but different governance approaches led to a different COVID-19 outcomes, including high mortality rates during the transition phases and the subsequent waves.

## Introduction

The Covid-19 pandemic has affected every country across the world, reaching a total of 130 165 012 positive cases by end of March 2021.^1^ The three small independent islands of the Republic of Cyprus, Iceland and Malta in Europe are no exceptions. Both the Republic of Cyprus and Malta are found within the Mediterranean Sea, having a total population of 855 900 and 442 429, respectively. Iceland is situated between the North Atlantic and Arctic Oceans with a total population of 364 134. Out of the three islands, Malta has the most ageing population with 15.8% of the total population above 64 years of age, as opposed to Cyprus (10.4%) and Iceland (12.7%).^2^ The life expectancy for Cyprus is 77.8 years (male 75.0, female 80.7), for Iceland is 80.9 years (male 78.7, female 83.2) and for Malta is 79.7 years (male 77.5, female 82.1).^2^ Malta has the worse metabolic profile out of the three islands, with 34.1% of the population obese (2014–16) and 10.4% with type 2 diabetes (2014–16) which is similar to Cyprus.^3^ The obesity rate in Cyprus was reported at 27.8% (2021), while in Iceland reported at 26.6% (2017).^3^^,^^4^

Like the rest of the countries in Europe, these three island states experienced a Covid-19 viral spread among their population at the beginning of 2020. All three islands had pandemic preparedness plans prior to the Covid-19 outbreak, with Malta starting as early as January 2020.^5^ These Islands having no land boarders and with a relatively small population, it was expected that infectious disease containment should follow similar trends while exhibiting a good containment outcome. The aim of this study was to compare the Covid-19 situation, restrictions, preparedness, management, vaccination and the healthcare systems after a year of Covid-19 between the small population island states of Cyprus, Iceland and Malta.

## Methods

Data were obtained from each country’s Ministry of Health websites, Covid-19 dashboards and locally related published studies. These datasets form part of the official public health trace-track surveillance and reporting system of each country on Covid-19.^6–8^ Excess deaths data was obtained from Eurostat, where excess mortality represents the percentage of mortality per month compared to the monthly average mortality in 2016–19.^9^ Comparisons were made between the reported cases, deaths, excess deaths, swabbing rates, restrictions and mitigation measures, vaccination roll-out strategies and healthcare system structures. Furthermore, comparisons of the Covid-19 situation with larger neighbor countries were performed as follows: Greece for Cyprus, Norway for Iceland and Italy for Malta. Data for this cross-countries comparison was obtained from the European Centre for Disease Prevention and Control (ECDC) for the period between 6 January 2020 and 4 April 2021.^10^ No distinction was made between individuals dying with Covid-19 from individuals dying due to Covid-19, due to the lack of such data for Iceland and Malta.

The 2019 all-cause mortality and Years of Life Lost (YLL) ranking for each Island was obtained from the Global Burden of Disease Study.^11^ The YLL is a metric used in population health to measure the number of years lost due to premature death from a particular cause. This metric is calculated by identifying the number of deaths in an age group and multiplying it by a standard life expectancy for that age group. The World Health Organization life expectancy age groups tables for each country were used for this analysis.^12^ The mortality cases by age groups and gender were obtained from the respective Islands’ Covid-19 dashboards. The Covid-19 YLLs for each Island were compared to the YLLs ranking for leading 20 injuries, communicable and non-communicable diseases for each country.

## Results

### Covid-19 situation in Cyprus, Iceland and Malta

**Figure 1:**
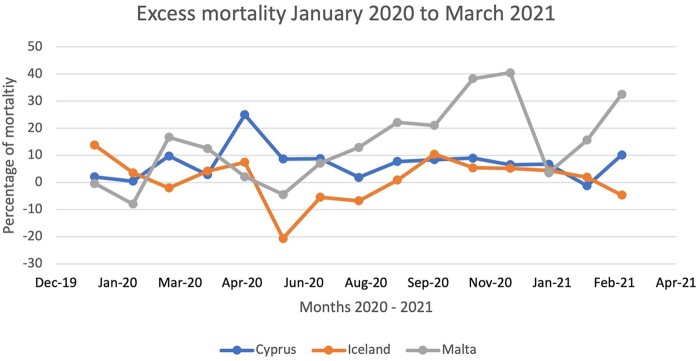
Comparison of the excess mortality for Cyprus, Iceland and Malta over a year

**Figure 2:**
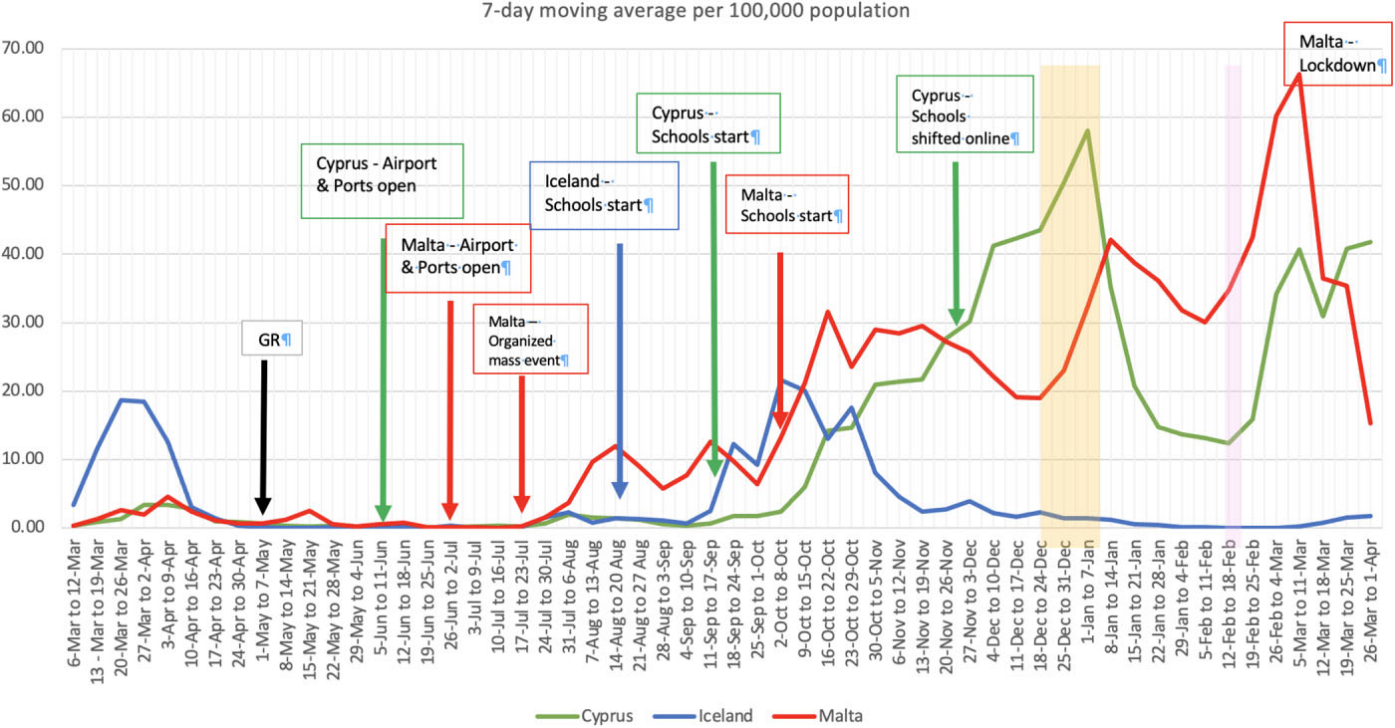
Comparison of the COVID-19 7-day moving average of positive cases and instituted measures/relaxations in Cyprus, Iceland and Malta over a year. GR – gradual relaxation of measures across the three Islands; Cyprus – opens airports and ports on 9th of June 2020; Malta – opens airport and ports on 1st July 2020; Iceland – new scholastic year started on 14th August 2020; Cyprus – new scholastic year started on 13th September 2020; Malta – new scholastic year started on 7th October 2020; Cyprus – schools shifted to online on 27th November 2020; Yellow shading – Christmas-New Year period; Pink shading – Carnival period in Malta

Iceland reported the first positive case on the 28th of February 2020 followed by Malta on the 7th of March and Cyprus on the 9 March 2020.^5^^,^^13^^,^^14^ The first Covid-19 related death was reported in Cyprus 11 days (21st of March) after the Covid-19 onset on the island, while both Iceland (23rd March) and Malta (8th April) reported their first death approximately a month after the onset of Covid-19 in these islands, respectively.^5^^,^^13^^,^^14^ Up until the 31st of March 2021, the total reported Covid-19 positive cases across the islands, per 100 000 people, were 5,330 Cypriots, 1,496 Icelandic and 6,563 Maltese, while the total Covid-19 deaths per 100 000 were 29 deaths for Cyprus (2.85% of all-cause mortality), 8 deaths for Iceland (1.37% of all-cause mortality) and 89 deaths for Malta (10.42% of all cause-mortality).^7^^,^^13^^,^^14^Figure 1 illustrates the excess mortality for the three islands between January 2020 to March 2021. Overall, Malta appeared to have reported the highest excess mortality rate, with a dip around January 2021 in keeping with other larger countries.^9^Figure 2 illustrates the 7-day moving average of positive cases along with the major instituted measures and relaxations across the three islands, over a year of the Covid-19 pandemic. During the first Covid-19 wave (March to 1st week of May 2020), a clear difference in the viral spread could be observed between Iceland and the other two islands. Both Cyprus and Malta (cumulative positive cases 104 per 100 000 and 109 per 100 000, respectively) exhibited a well-controlled situation with low daily case numbers when compared to Iceland (cumulative positive cases 497 per 100 000).^5^ This was attributed to the return of Covid-19 infected Icelanders from skiing holidays resulting in high population transmission before the government instituted restrictive measures including quarantine to inbound passengers.^15^ However, a different scenario can be observed for the second Covid-19 wave, as shown in figure 2, with Malta entering the second wave at a much earlier phase than the other Islands as well as having the highest total positive cases over a year.^16^

On comparing the Covid-19 cases of the three small islands to the larger neighboring countries, it was noted that the global cumulative positive cases per 100 000 population (until 4th April 2021) for Cyprus (4,692.99), Iceland (1,746.88) and Malta (5,680.93) were lower than their neighboring countries of Greece (5,1001.56), Norway (1,853.63) and Italy (6,185.50), respectively.

### COVID-19 first wave: restrictions and legislations

A number of similar restrictions and mandatory legislations were instituted during the first Covid-19 wave by the three small islands’ governments, as seen in Supplementary table S1, with a Public Health Emergency declared by each country.^5^^,^^8^^,^^14^^,^^17^ Concurrently, a number of economic stimulus packages and benefits were implemented by all governments to aid struggling businesses.^5^^,^^8^^,^^17^ The implementation of these restrictions and measures along with the population adherence to personal hygiene, restrictive traveling and social distances led to an abrupt decrease in Covid-19 cases across the Islands as shown in figure 2.

**Table 1:** Comparative Covid-19 vaccination statistics across Cyprus, Iceland and Malta^32–35^

		Cyprus	Iceland	Malta
Vaccination		**Until 17th April**	**Until 21st April 2021**	**Until 20th April 2021**
	Fully vaccinated	6773 per 10 000	8453 per 100 000	20 844 per 100 000
	One dose	178 300 per 10 000	18 062 per 100 000	44 912 per 100 000
Vaccines available				
	Pfizer-BioNTech	Yes from 27 December 2020	Yes from 29 December 2020	Yes from 27 December 2020
	Moderna	Yes	Yes from 13 January 2021	Yes from 11 January 2021
	Oxford/AstraZenea	Yes	Yes from 11 February 2021	Yes from 14 February 2021

### Transition phase and the emergence of the second and consecutive waves

Similar to the other countries in Europe, the first wave restrictions including travel bans began to be eased in May–July 2020, with the anticipation of a ‘new normal’ while slowly restarting the national economies.^18^^,^^19^ Such actions were expected to invoke a high risk of viral resurgence with a balancing act between stimulating the economy and minimizing morbidity/mortality from Covid-19.^20^^,^^21^ In the initial phases of the transition phase, the Republic of Cyprus was observed to have had the best curbing ability of the viral infection out of the three small islands as shown in figure 2. However, a higher spike could be observed just 10 days after school opened on the 13th of September 2020. Meanwhile, Iceland’s second wave was reported at the end of July 2020 with speculations that a football game was the triggering factor.^22^ Concurrently, new restrictions were introduced by all three Islands’ governments to try to curb this new wave. The re-introduced restrictions appeared to curb the spread in Iceland.^23^ Indeed, on the 7th of September some measures in Iceland were relaxed. However, a significant spike in positive cases could be observed ten days following the relaxation of these measures, as shown in figure 2. A different scenario could be observed for the island of Malta, where the viral spread was well controlled up until mid-July where organized mass events took place mid-July 2020, resulting in a significantly spike of new positive cases (figure 2) and high community spread.^16^ The re-introduction of restrictions, led to a slight reduction in daily cases in Malta; however, the spread within the community was still high, with a significant spill off to elderly nursing homes.^7^^,^^16^ Consequently, another high spike in positive cases and 14 deaths in just a week, was observed among the Maltese elderly population.^7^

**Figure 3:**
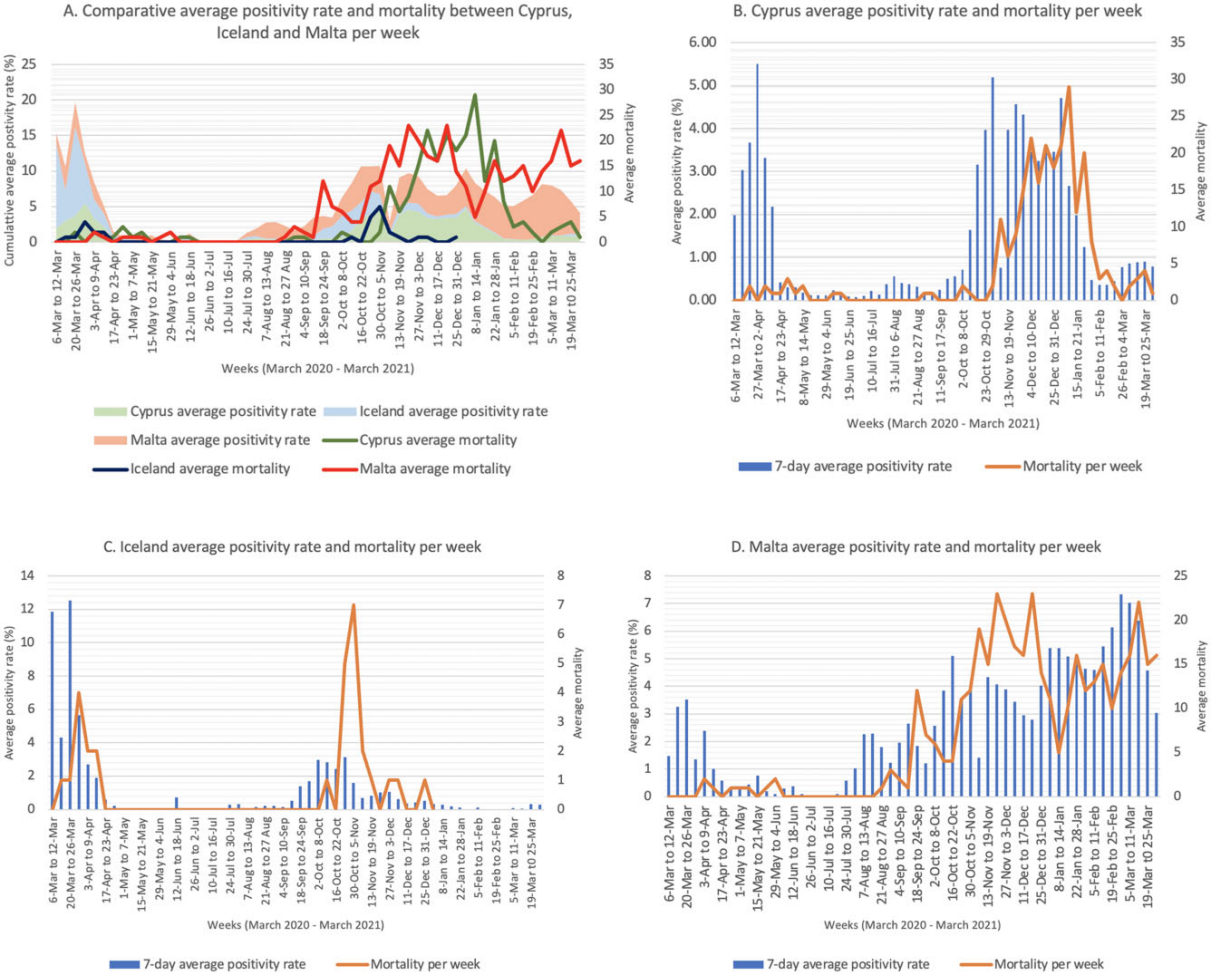
Comparison between positivity rate moving average and mortality rate per week (March 2020 to March 2021). (A) Comparisons between Cyprus, Iceland and Malta; (B) Comparison for Cyprus; (C) Comparison for Iceland; (D) Comparison for Malta.

The positivity rate (number of positive cases out of total swabs per day) could be observed to fluctuate across the Covid-19 year for the three islands with a consequential increase in mortality rate as shown in figure 3. The average positivity rate across the Covid-19 year was 1.59% for Cyprus, 1.35% for Iceland and 2.71% for Malta.^7^^,^^13^^,^^14^ Both Cyprus and Malta exhibited high positivity rates by end of Summer 2020, however Malta experienced a much consequential higher mortality rate, as well as continued with high weekly positivity rates up till March 2021 (figure 3). On the other hand, Iceland was observed to have had a better control over the viral spread and mortality rate than the other two islands (figure 3). Indeed, no mortality cases were reported as of January 2021 in Iceland.

On measuring the annual Covid-19 YLL for each Island, Malta exhibited the highest YLL (4129), followed by Cyprus (2474) and Iceland (301). Each island’s Covid-19 YLL was compared to the leading 20 ranking injuries, communicable and non-communicable diseases YLLs for each Island, as reported by the Global Burden of Disease Study (2019).^11^ For the Covid-19 YLL, in Malta ranked with the third highest YLL, following stroke (YLL =4282) and ischemic heart disease (YLL = 12 998), while Cyprus ranked at the 16th position following injuries and several non-communicable diseases. Meanwhile, the Covid-19 YLL for Iceland did not rank within the leading 20 YLLs.

### Healthcare systems and services

Following the onset of SARS-CoV-2 in China in December 2019^24^ all the three small Islands commenced preparations for the inevitable Covid-19 outbreaks in their countries. Both Iceland and Malta purchased a substantial amount of medical supplies in advance to ensure an adequate supply for pandemic peak.^5^^,^^25^ Conversely, a shortage of medical supplies, especially surgical masks, was evident in Cyprus in early April, leading to a high infection rate among Cypriot healthcare professionals.

In Cyprus, the public Ammochostos General Hospital (AGH) was appointed to be the Covid-19 reference point in December 2019 by the Ministry of Health. The Intensive Care Unit (ICU) of the AGH was equipped with ventilators. In Cyprus an ICU in Nicosia General Hospital (NGH) was built for the same purposes in case of a high number of Covid-19 cases.^26^

In Iceland, the two largest hospitals were designated to treat all Covid-19 cases, with special Covid-19 wards created and staff relocated from different services within each hospital to work in these wards. Infrastructural changes were made to Iceland’s and Malta’s state hospitals, with an increase in intensive care unit (ICU) beds and ventilators to accommodate potential Covid-19 surges in critical cases.^5^^,^^25^^,^^27^

In Malta, there is only one acute state hospital to serve the whole island, which had designated wards and areas equipped for Covid-19. A number of non-clinical areas within the hospital building (such as the Medical School library, lecture rooms and staff canteen) underwent infrastructural changes and were converted into temporary wards.^25^^,^^27^ Malta’s hospital public areas (such as hospital’s foyer and outpatient’s corridors) were also equipped with oxygen points in preparation for a potential need to increase the hospital beds.^27^

Across the three islands, outpatient clinics and elective surgery were suspended (March to May 2020) in order to ensure that an adequate number of available beds were present for potential COVID-19 cases while simultaneously increasing the number of healthcare professionals to form part of the COVID-19 the management team.^5^^,^^8^

Concurrently, Public Health authorities established a crisis management contact tracing unit and case management teams to trace the positive cases contacts. In Malta the public health specialists were responsible for the contact tracing, swabbing and surveillance. While, the primary healthcare physicians were robbed in to follow up the clinical situation of Covid-19 positive cases quarantined at their own home. In Cyprus, an Organized unit of the Ministry of Health was set-up in early March 2020 and was responsible for the tracing of positive cases. The KIOS (Research and Innovation Center of Excellence) platform under the aegis of the Ministry of Research, Innovation and Digital Policy was set-up in collaboration with the University of Cyprus.^28^ The platform provides information with daily updates on the Covid-19 cases and later provided also vaccination information (https://covid19.ucy.ac.cy/). Additionally, a volunteering team was set-up to deal with the excessive number of emergency calls made to the ambulance unit. Both in Iceland and in Malta a digital health information platform was created to provide updated information on the Covid-19 situation as well as advise.^7^^,^^29^ In Malta, all restrictions and legislations instituted were published on this digital platform, in addition to the daily Covid-19 infographics.

### Swabbing for SARS-CoV2

A high daily swabbing rate was conducted across all the small Islands. Cyprus conducted 405 286 swabs per 100 000 population, Iceland conducted 149 809 swabs per 100 000 population while Malta conducted 183 132 swabs per 100 000 population by the end of March 2021.^7^^,^^13^^,^^14^ Several swabbing hubs were set up across the islands for easy accessibility by the served populations. More information on the swabbing testing procedures for each Island can be found in the Supplementary material.

### Cross border restrictions

As of 15th of June 2020, those arriving to Iceland were mandated to either take a Covid-19 swab test at the border or quarantine for 14 days. Later on, as of 19th of August, anyone arriving to Iceland had to undergo two border COVID-19 swab tests 5 days apart unless they opted for a 14-day quarantine.^14^ Initially in Malta, as airport opened on 1st of July 2020, a random Covid-19 swab test approach was introduced. On the 17th of August 2020, Malta introduced an ‘Amber country list’ where travelers arriving from these high-risk countries needed to represent a negative Covid-19 test conducted in the past 72 h. If this was not available, a swab test was performed at the airport.^16^ Travelers from ‘Red country list’ were banned to travel to Malta unless they were citizens or had a work permit, in which case they needed to quarantine. As of the 31st of March 2021, anyone arriving in Malta needed to present a PCR swab test or else stay in quarantine.^30^ On re-opening the airports in June 2020, Cyprus offered Covid-19 PCR tests for free to tourists arriving on the Island. However, this was re-visited later to mandate all arrivals to present a negative test done within the past 72 h.

### Covid-19 vaccination

Each of the three Islands’ governments formulated a national vaccination strategy plan to vaccinate the population in a prioritization order. Across the three Islands, the first vaccine to arrive was the Pfizer-BioNTech vaccine in December 2020 (table 1). Malta is currently the leading European country in vaccination, with 18.06% of the population fully vaccinated and 41.87% taken the first dose (up till 13th April 2020).^7^^,^^31^ The Ministry of Health of the Republic of Cyprus issued a national vaccination plan on the 15th of December 2020 with the optimistic goal of providing vaccination coverage to more than 40% of the Cyprus citizens during the first 6 months of 2021.^32^

## Discussion

Small states and islands are known to face several challenges. However, when controlling infectious diseases their small population and geographical size plays to their strengths. Islands have another advantage with the absence of land borders, making containment of infectious disease easier if effective and timely restrictions are implemented. During the first Covid-19 wave (March–June 2020), the small islands of Malta and Cyprus appeared to have had the upper hand in controlling the Covid-19 spread compared to Iceland despite similar containment measures (with some exceptions) instituted across the three islands. Differences could be observed with regards to the closure and re-opening of airports regulations. Iceland never officially closed the airport; in fact, a higher positivity rate was observed in Iceland when compared to the other two islands. However, but when other countries started to re-open their airports, Iceland implemented a thorough border system to ensure Covid-19 is not imported to the community. The re-opening of airports in Cyprus did not lead to any spikes in positive cases and this was later enforced by a mandatory Covid-19 negative test for all arrivals, a measure not implemented in Malta in Summer 2020. This does not preclude the fact that cross-border control is essential to ensure the safety and reduced transmissibility across countries. Indeed, when borders were closed in both Cyprus and Malta, both populations experienced the lowest transmissibility and mortality rates when compared to the succeeding waves.

The triggering factor for the onset of the second wave in Malta does not appear to have been the re-opening of the airport or port but rather the unrestrictive capping of people gathered in one place^16^ unlike in Cyprus and Iceland. Despite the re-introduction of restrictions, Malta continued to struggle with high community spread as schools opened and following the festive periods of Christmas, New Year and the Carnival. The second wave for Cyprus was triggered in late September with the re-opening of schools, errors in shielding nursing homes in October and the re-opening of catering areas. Further restrictions were imposed in November to control the spread, only temporarily. The infection rates continued to rise, shopping malls and entertainment areas continued to operate, until further restrictions posed in December in a desperate attempt to ‘save the Christmas holidays’. As expected, Cyprus experienced an inclined peak in positive cases during the Christmas–New Year festive period, after which the restrictive measures appeared to have brought down the daily counts and mortality rates. This was only the continuation however, of a worse and prolonged wave of infections lasting until the mid-February. Then a third wave appeared to be even bigger and possibly longer as to date is still on-going, and with the Covid-19 reference point hospital operating at 98% of its capacity.

Out of the three islands, Iceland appears to have had the best Covid-19 outcome in the past year, with the lowest Covid-19 infected population, low daily case numbers were reported from December 2020 until March 2021 as well as no Covid-19 related mortality cases from January 2021.^14^

It is evident that when restrictive measures were implemented including closing of borders, restriction of movement, the Covid-19 transmission and mortality was controlled. The opposite occurred when measures were relaxed. In which case, the upper hand an island state exhibited diminished. However, all three islands still exhibited a lower Covid-19 cumulative infectivity when compared to their neighboring larger countries, which suggest that an element of advantage was still present in being an island state.

## Conclusion

The small population size and island status proved to be an asset during the first wave of Covid-19 but different governance approaches led to a different Covid-19 outcome during the transition phase, the second and consecutive waves. Timely public health action including cross-border restrictions along with restrictions on gatherings and school closure should be implemented if the need arises, along with continuous surveillance of the situation. It is paramount to learn from past experiences and ensure that future policies reflect these. Digital health information should play a part in public awareness, advice and guidance including the importance of social distancing and personal hygiene, mandatory wearing of masks while enhancing the Covid-19 vaccination program roll-out.

## Supplementary data

Supplementary data are available at *EURPUB* online.

**Key** **points**

- Small population size and island status proved to be an asset during the first COVID-19 wave.
- Different governance approaches led to different COVID-19 situations even among small populations.
- Restrictions in the number of people gathered together is an essential mitigation measure.
- Controlling the positivity rate is essential irrelevant of the geographical population size

## Supplementary Material

ckab217_Supplementary_Data
