# Supplementary material for: A year of Covid-19: experiences and lessons learnt by small European island states—Cyprus, Iceland and Malta
Source: Eur J Public Health. 2022 Jan 3;32(2):316–21. doi: 10.1093/eurpub/ckab217 (PMC8755372; doi:10.1093/eurpub/ckab217)
Supplement: ckab217_Supplementary_Data [file ckab217_supplementary_data.zip › ckab217-suppl_data/ejph-2021-04-om-0522-File006.docx]

Supplement material:

Swabbing for SARS-CoV2 across the three islands

Both Iceland and Malta followed a similar swabbing test procedure, where symptomatic and concerned individuals called a designated helpline to organize a swabbing test. Later, Malta set up a designated digital portal to book a swabbing test. Only appointment-based swabs were carried out. In Iceland, only those with symptoms were offered the swab test for free, while all others, including tests done at the border, cost between €55 to €68 (9000 – 11000 ISK). On the other hand, in Malta all swabs were done for free unless the individual opted to perform the swab test at a private hospital. In Cyprus, the Ministry of Health has been issuing decrees, calling different working groups to be tested on different dates. All other individuals opting to have the swab test could visit a swabbing hub and pay for the service (initially €110 and reduced to €60 in July 2020). From November onwards (until April 2021 when this paper was written), another decree determined that all workers had to have a valid test on a weekly basis to be shown to an MoH representative presentative when requested.

All countries provided the swab test results within a 24-hour period while the individual remained under quarantine until the result was provided through a mobile text or phone call by the public health authorities (applicable for positive cases in Malta). Penalties were instituted by governments within all three small Islands for those breaching the mandatory quarantine. In Cyprus those breaching these restrictions were taken to court with a possible jail sentence up to six months, payment of all legal expenses and fined €768 per individual. In Iceland, those breaching or refusing the mandatory quarantine were fined between €310 to €1,549 (50,000 – 250,000 ISK). Similarly, in Malta those in breach of mandatory quarantine were fined €10,000 while those ordered to stay in quarantine due to having had a contact with a positive cases were fined €3,000 ^1^.

References:

1. Cuschieri S. COVID-19 panic, solidarity and equity—the Malta exemplary experience. *J Public Heal*. 2020;30:1-6. doi:10.1007/s10389-020-01308-w
