# Supplementary material for: A year of Covid-19: experiences and lessons learnt by small European island states—Cyprus, Iceland and Malta
Source: Eur J Public Health. 2022 Jan 3;32(2):316–21. doi: 10.1093/eurpub/ckab217 (PMC8755372; doi:10.1093/eurpub/ckab217)
Supplement: ckab217_Supplementary_Data [file ckab217_supplementary_data.zip › ckab217-suppl_data/ejph-2021-04-om-0522-File007.docx]

|  | **First wave** | | |
| --- | --- | --- | --- |
| **Restrictions and Mandates** | **Cyprus** | **Iceland** | **Malta** |
| Quarantine for 14 days for arriving passengers | Yes | Yes | Yes |
| Closure of Airport | Yes | No but reduced schedule | Yes |
| Closure of Ports | Yes | No but reduced freight | Yes |
| Permitted number of people in one Gathering | Capped | Capped to 100 (16/03/2020)  then reduced to 20 (23/03/2020) | Capped to 3 (02/04/2020) |
| Social distancing | Yes | Yes | Yes |
| Face to face education at schools | Closed | Closure of universities and high schools.  Preschool, elementary schools remained open | Closed |
| Non-essential retails | Closed | Closed | Closed |
| Bars and restaurants | Closed | Closed | Closed |
| Vulnerable Groups Act | Yes | Yes | Yes |
| Masks | Recommended | Not recommended | Recommended |

Supplement Table 1. Comparisons between the restrictions and mandatory legislations instituted by the three small Islands during the first COVID-19 wave ^4,6,9,10^
